# From infestation to infection: a systematic review of arthropod-mediated microbial transmission in hospitals

**DOI:** 10.1017/ash.2025.10266

**Published:** 2026-01-12

**Authors:** Stephanie Stroever, Rishi Patel, Barrett Meeks, Nicholas Schouten, Dan Stuart, Jennifer Hanrahan, Kendra Rumbaugh, Stephen P. Diggle

**Affiliations:** 1 Department of Medical Education, School of Medicine, Texas Tech University Health Sciences Center, Lubbock, TX, USA; 2 School of Medicine, Texas Tech University Health Sciences Center, Amarillo, TX, USA; 3 University of Texas Southwestern Medical Center, Dallas, TX, USA; 4 Joan C. Edwards School of Medicine. Marshall University, Huntington, WV, USA; 5 Department of Surgery, School of Medicine, Texas Tech University Health Sciences Center, Lubbock, TX, USA; 6 School of Biological Sciences, Georgia Institute of Technology, Atlanta, GA, USA

## Abstract

**Objectives:**

To evaluate the evidence for arthropod-associated transmission of pathogens in healthcare facilities and synthesize available literature to support infection prevention practice and policy.

**Methods:**

We conducted a systematic review following PRISMA guidelines. PubMed, Ovid MEDLINE, Embase, and CENTRAL were searched using terms related to insects/arthropods, microbial transmission, and healthcare-associated infection, supplemented by grey literature and citation tracking. No date, language, or study-type restrictions were applied. After deduplication and screening in Covidence, 73 studies met inclusion criteria. Eligible studies described microbial isolation or transmission involving arthropods within healthcare environments. Data were extracted by multiple reviewers using a standardized manual and verified by a senior author. Analyses were descriptive and focused on identifying evidence of patient transmission and microbial carriage among arthropods.

**Results:**

Included studies spanned all world regions, most commonly from Asia and South America. Cockroaches, flies, and ants were the primary arthropods examined, with samples collected from both clinical and non-clinical hospital areas. Six studies investigated direct patient transmission, and three provided genetic or circumstantial evidence linking arthropods to patient infections, including *Klebsiella pneumoniae*, multidrug-resistant *Enterobacter cloacae*, and dengue virus. The majority of studies identified arthropods carrying clinically significant bacteria (eg, *Staphylococcus aureus*, *Enterococcus faecalis*, *Acinetobacter baumannii*, *Pseudomonas aeruginosa*), fungi (*Aspergillus, Candida*), and viruses.

**Conclusions:**

Arthropods in healthcare environments commonly harbor clinically relevant and antimicrobial-resistant microbes, and limited but compelling evidence supports their potential role in patient transmission. Strengthened pest management and environmental hygiene are essential components of infection prevention.

## Introduction

Arthropods are ubiquitous in the global ecosystem, and while primarily outdoors, arthropods enter and persist within human-built structures, including healthcare facilities. Reports from multiple countries describe infestation of arthropods in the healthcare environment (HE) involving arthropods (ants, cockroaches, flies, beetles) and arachnids (spiders and scorpions).^1–3^ This raises concerns about their potential role in the transmission of pathogens to patients.

Infection prevention (IP) programs play a central role in identifying and mitigating risks to patients, including those associated with pests. Despite integrated pest management programs (IPMs), arthropods remain challenging.^4,5^ Although intuitive that arthropods may pose a threat, the extent is not well defined despite three decades of research. For instance, if arthropods contaminate sterile storage areas or encounter medical supplies, does this translate into indirect transmission to patients? And if so, to what degree? This uncertainty has important implications: when infestation occurs, IPs must be able to make evidence-based recommendations about whether supplies should be reprocessed, discarded, or remain in use. Federal regulations reflect this expectation: the Centers for Medicare and Medicaid Services (CMS) require hospitals to maintain premises free from arthropods and rodents through an IPM (42 CFR 485.725) and to ensure IPs maintain a sanitary environment that minimizes infection risk (42 CFR 482.42).^6,7^

Documented cases of myiasis in hospital environments further illustrate the potential for arthropods to compromise patient safety. In one report, a patient recovering in an intensive care unit (ICU) developed traumatic myiasis with *Sarcophaga* species.^8^ Despite wound dressings, the flies gained access beneath dressings during recovery. Such events highlight the ability of arthropods to infiltrate tight spaces and reinforce the concern that their presence in HE may present transmission risks.

Several systematic reviews have examined the role of arthropods in HE, typically focusing on carriage of pathogens.^9–11^ These reviews are often limited to a single type of arthropod, such as flies or cockroaches, and largely describe the isolation of pathogens from arthropod surfaces or gastrointestinal tracts. While these findings establish that arthropods can harbor potentially pathogenic microbes, they do not directly address transmission. This distinction is critical: carriage demonstrates potential, but only transmission demonstrates actual risk.

The primary objective of this study was to determine the extent to which the literature demonstrates transmission of pathogens to patients in HE via arthropods. The secondary objective was to develop a comprehensive accounting of available literature to provide a single source of evidence for IPs to use for guidance.

## Methods

### Search strategy

We conducted a systematic literature search using research databases and information resources in accordance with PRISMA recommendations. PubMed, Ovid MEDLINE, Embase, and the Cochrane Register of Controlled Trials (CENTRAL) were searched using a reproducible strategy comprised of preferred indexing terms associated with “insects,” “microbial transmissions,” “insect vector,” and “cross infection” (Appendix 1). Further studies were identified in ClinicalTrials.gov and Google Scholar. Additional hand-searching and citation analysis using key foundational articles provided further records. Over 1,000 records were identified. These were deduplicated using Covidence, after which 655 eligible articles were screened, reviewed, and 73 were included for analysis.

### Eligibility, screening, and selection

Inclusion and exclusion criteria were established *a priori* and consulted during the screening and evaluation process. Targeted studies included articles reporting microbial transmission via arthropods in HEs. Excluded articles comprised infections with other identified sources. Review-based or non-primary study designs were also excluded. Two authors (RP, NS) independently assessed records for eligibility and evaluated full texts for inclusion. A third author (SS) resolved conflicts and applied consensus evaluations for data extraction.

### Data collection

All variables were extracted into a data collection form in Covidence. Four authors (RP, NS, JH, BM) extracted data after training using a shared manual of procedures developed with expert microbiologists (KR, SD). The senior author (SS) reviewed all forms for accuracy and consistency across extractors and studies flagged as unclear. Final versions were exported for synthesis.

The primary outcome was evidence of transmission of any microbe to a patient via an arthropod vector. Studies needed to demonstrate that a patient became either colonized or infected with a microorganism in the HE via arthropod. The secondary outcome was isolation of any pathogen from an arthropod in the HE.

Information collected included geographical location, HE type, arthropod type by both common and scientific names, and aims and objectives of each study.

Studies were classified by study design, whether they were conducted only in HE or had a non-healthcare control, the manner in which pathogens were cultured from the arthropod, and type of microbe studied.

The methods of microbial isolation and identification were also noted in the data collection form, as well as the type of analysis. Analysis was captured either as qualitative or quantitative. Combining the quantitative results into a meta-analysis was beyond the scope of this review. Finally, we described mechanisms of antimicrobial resistance identified in each study if reported.

### Data synthesis

All studies meeting inclusion criteria were included. Data synthesis was primarily descriptive.

## Results

### Study characteristics

**Figure 1.**
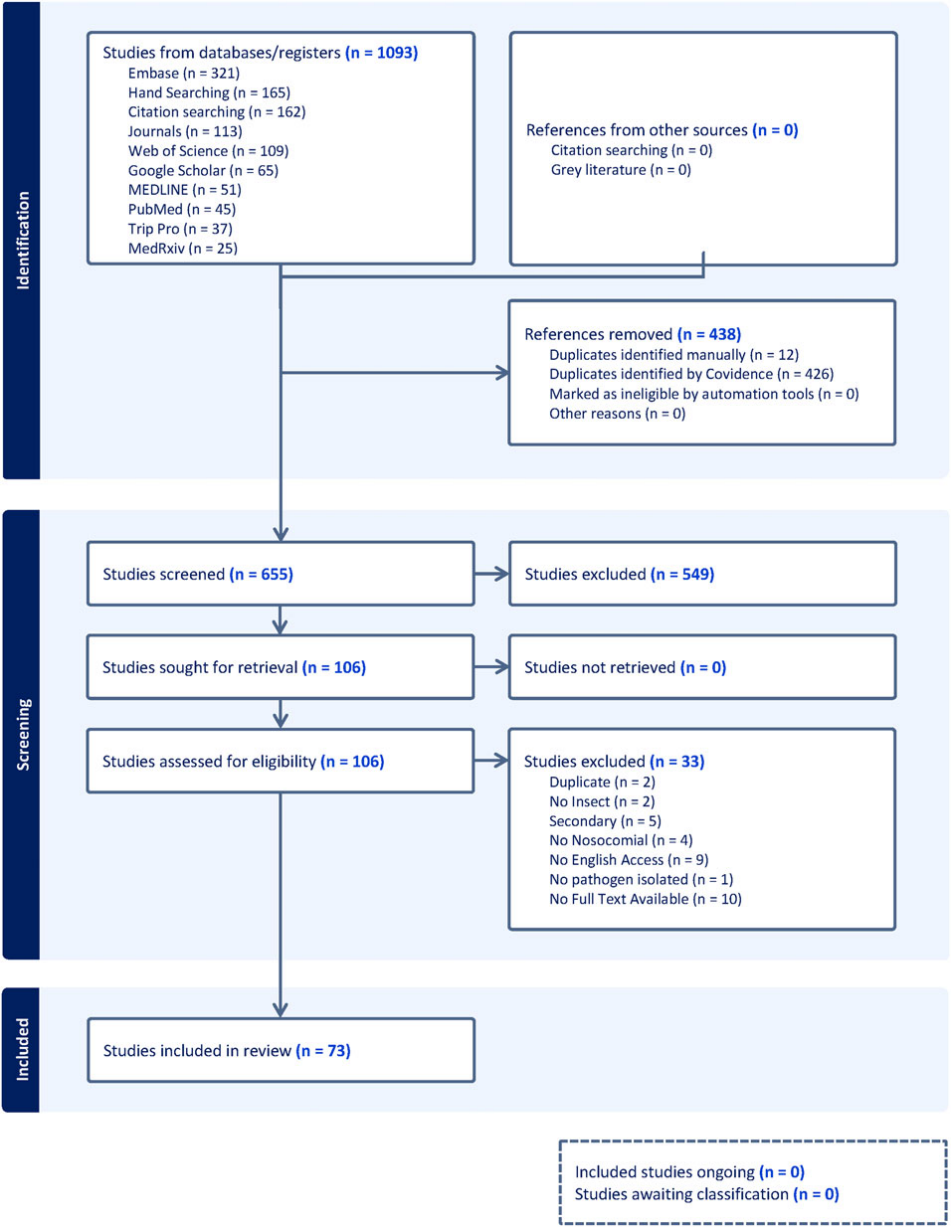
PRISMA diagram from Covidence reflecting identification, screening, and review process.

We included 73 studies. Figure 1 depicts the screening and selection process. Studies were conducted across all world regions, with the largest number originating from Asia and South America (Table 1). African and European countries were also represented, while relatively few studies came from North America. Within regions, studies tended to cluster in a handful of countries (eg, Brazil, Iran, and India), with only scattered contributions from others. Arthropods were collected in clinical and nonclinical areas of HEs including sewer systems (Table 1). About half of the studies focused on non-critical care or non-clinical areas, while a smaller proportion included critical care units. Many studies sampled arthropods from surrounding environments to compare with HE isolates.

**Table 1.** Overview of studies included in this systematic review examining arthropod-mediated microbial transmission in healthcare settings

			Setting					Arthropod				Microorganism			
Continent of study	Country of study	Study	Critical care units	Surgical or sterile areas	General medical or clinical units	Non-clinical areas	Community or external sites	Cockroach	Ant	Fly	Other	Gram-positive bacteria	Gram-negative bacteria	Fungi or yeast	Virus
Africa	Algeria	Loucif, 2016	+					+					+		
		Menasria, 2014		+		+		+				+	+		
		Mehainaoui, 2021			+			+				+	+		
		Merad, 2023			+	+		+						+	
	Ethiopia	Tilahun, 2012	+					+				+	+		
		Tufa, 2020	+		+					+		+	+		
	Libya	Elgderi, 2006			+		+	+				+	+		
		Rahuma, 2005			+	+	+			+		+	+		
	Kenya	Chiuya, 2021			+		+				+				+
	Nigeria	Adegoke, 2021		+	+		+	+				+			
	Tunisia	Landolsi, 2022			+	+	+	+				+	+		
	Rwanda	Heiden, 2020	+		+	+				+		+	+		
	South Africa	Monyama, 2023					+			+		+	+		
		Cotton, 2000	+		+			+					+		
Asia	Bangladesh	Sobur, 2022				+				+		+			
		Naher, 2018			+		+	+				+	+	+	
	India	Fotedar, 1992a			+		+	+		+			+		
		Fotedar, 1992b			+		+	+						+	
		Fotedar, 1992c			+		+	+		+		+	+	+	
		Fotedar, 1991a	+		+		+	+				+	+	+	
		Fotedar, 1991b			+		+	+					+		
	Iran	Davari, 2023					+	+		+					
		Madani, 2023				+		+						+	
		Abdolmaleki, 2019a^*^						+				+			
		Abdolmaleki, 2019b^*^						+				+			
		Kassiri, 2015			+			+		+					
		Fakoorziba, 2014						+				+	+		
		Nazari, 2017				+	+			+		+	+		
		Ranjbar, 2016					+			+			+		
		Khodabandeh, 2020					+	+						+	
		Chehelgerdi, 2021^*^						+							
		Kassiri, 2014			+	+		+				+	+	+	
		Davari, 2012					+	+		+		+	+	+	
		Zarchi, 2009				+		+				+	+		
		Salehzadeh, 2007			+	+	+	+				+	+	+	
	Japan	Watanabe, 2019			+						+	+			
	Sri Lanka	Ehelepola, 2018^*^								+					+
	Taiwan	Pai, 2003			+	+	+	+					+		
		Pai, 2004			+	+		+				+	+	+	
	Pakistan	Memona, 2017	+		+	+	+	+				+	+		
		Hassan, 2021									+		+		
	Iraq	Jalil, 2023				+	+	+				+	+		
Europe	Poland	Wiktorczyk-Kapischke, 2022					+			+		+	+		
		Gliniewicz, 2003			+			+				+	+		
		Stypułkowska-Misiurewicz, 2006		+	+			+				+	+		
	United Kingdom	Davies, 2014^*^								+		+	+		
		Beatson, 1972	+		+	+			+			+	+		
		Boiocchi, 2019	+		+					+		+	+		
	Czech Republic	Sràmovà, 1992		+	+	+	+				+	+	+	+	
		Daniel, 1992		+	+	+	+				+	+	+	+	
	France	LeGuyader, 1989			+	+		+				+	+		
	Germany	Faulde, 2012			+	+				+		+	+		
		Rupprecht, 2020	+	+	+	+				+		+	+		
		Faulde, 2001			+					+		+	+	+	
		Frickmann, 2024			+				+			+	+	+	
South America	Brazil	Lise, 2006			+				+			+	+		
		DoNascimento, 2020	+		+	+			+			+	+		
		Máximo, 2014	+	+	+	+	+		+			+	+		
		Oliveira, 2017	+	+	+	+					+	+	+		
		Aquino, 2013	+		+	+			+					+	
		Carramaschi, 2019				+	+			+			+		
		Prado, 2006			+	+		+				+	+		
		Krokovsky, 2022			+						+				+
		Lemos, 2006	+					+						+	
		Rodovalho, 2007	+		+		+		+			+	+		
		Kappel, 2013	+		+						+	+	+		
		Lima, 2013	+	+	+	+	+		+			+	+		
		Oliveira, 2014		+	+	+			+		+	+			
		Almeida-Nunes, 2016	+		+						+				+
	Columbia	Rodríguez, 2016	+			+			+			+	+		
North America	United States	Hanrahan, 2024	+					+				+	+		
	Cuba	Oliva, 2010^*^						+				+	+	+	
	Trinidad, West Indies	Chadee, 1990	+		+	+			+			+	+		

Note. Critical care units include adult intensive care units (surgical, medical), neonatal intensive care unit, oncology unit, burn unit; surgical or sterile areas include sterile procedure rooms, operating rooms, sterile storage; General medical or clinical units includes medical/surgical wards, emergency room, outpatient clinics (including dental); non-clinical areas include cafeteria, kitchen, hallways, waiting rooms, loading docks, and hospital sewer; community or external sites includes any space or facility external to a healthcare facility; laboratory includes the study conducted in the lab.

* Hospital or clinical unit not specifically named (eg, hospital).

Cockroaches, flies, and ants were among the most common arthropods studied, with additional investigations involving beetles, moths, wasps, and mosquitoes (Supplemental Table 1). A variety of strategies were used to collect arthropods and identify pathogens (Table 2). Most used a cross-sectional design, sampling arthropods continuously over a defined period but not using longitudinal methods to demonstrate temporal changes. Standard approaches to isolate microbes included external washing, dissection, and culturing on selective media, although methods varied by arthropod type (eg, whole-body homogenization for flies, swabbing, or broth enrichment for ants). Microbial identification commonly relied on culture-based techniques, supplemented in some studies with molecular methods. Antimicrobial resistance testing was frequently performed.

**Table 2.** Methods used to isolate and identify microorganisms from captured arthropods in this systematic review

	Microbial extraction		Microbial isolation and growth		Microbial identification					
Study	External washing of arthropod/collection of excrement	Dissection of internal contents of arthropod	Culture with selective media	Other	Biochemical profile with Gram stain and morphology	Antimicrobial sensitivity testing	MALDI-TOF MS	Pulsed-field gel electrophoresis	16S rRNA sequencing	Other
Abdolmaleki, 2019a	+	+	+			+		+		+
Abdolmaleki, 2019b	+		+			+		+		+
Adegoke, 2021	+		+			+				
Almeida-Nunes, 2016				+						+
Aquino, 2013	+		+		+					+
Beatson, 1972	+		+							
Boiocchi, 2019	+	+	+			+				
Carramaschi, 2019		+					+		+	+
Chadee, 1990^*^										
Chehelgerdi, 2021	+		+		+	+		+	+	
Chiuya, 2021		+						+	+	
Cotton, 2000	+		+		+			+		+
Daniel, 1992	+		+		+	+				+
Davari, 2012	+		+	+						+
Davari, 2023	+		+		+	+				
Davies, 2014	+	+	+		+					
DoNascimento, 2020	+		+		+					
Ehelepola, 2018			+							+
Elgderi, 2006	+	+	+			+				
Fakoorziba, 2014	+	+	+		+	+				
Faulde, 2001	+				+					
Faulde, 2012	+	+	+		+	+				
Fotedar, 1991a	+	+	+		+	+				
Fotedar, 1991b	+	+	+		+	+				
Fotedar, 1992a	+	+	+		+	+				
Fotedar, 1992b	+	+	+							
Fotedar, 1992c	+	+	+		+	+				
Frickmann, 2024	+	+	+		+		+			
Gliniewicz, 2003	+		+		+	+				
Hanrahan, 2024	+		+		+	+		+		
Hassan, 2021^*^			+			+	+			+
Heiden, 2020^*^			+		+	+	+			
Jalil, 2023	+		+		+	+				
Kappel, 2013	+		+		+					
Kassiri, 2014	+		+		+					
Kassiri, 2015	+	+	+		+					
Khodabandeh, 2020	+	+	+		+					
Krokovsky, 2022	+	+								+
Landolsi, 2022	+	+	+			+				+
LeGuyader, 1989	+	+	+		+					
Lemos, 2006	+		+		+					
Lima, 2013	+		+							
Lise, 2006	+	+	+		+	+				
Loucif, 2016	+	+	+		+	+	+			
Madani, 2023	+	+	+							+
Máximo, 2014	+		+		+					
Mehainaoui, 2021	+	+	+		+		+		+	
Memona, 2017	+	+	+		+					
Menasria, 2014	+	+	+		+					
Merad, 2023	+		+		+					
Monyama, 2023	+	+						+	+	+
Naher, 2018	+	+	+		+	+				
Nazari, 2017	+		+		+	+				
Oliva, 2010	+		+		+					
Oliveira, 2014	+		+		+	+		+		
Oliveira, 2017	+		+		+	+				
Pai, 2003	+	+	+		+	+				
Pai, 2004	+	+	+		+	+				
Prado, 2006	+	+	+		+	+				
Rahuma, 2005	+	+	+		+	+				
Ranjbar, 2016	+	+	+		+	+		+	+	
Rodovalho, 2007	+		+		+	+				
Rodríguez, 2016	+		+		+					
Rupprecht, 2020	+		+		+	+	+			
Salehzadeh, 2007	+	+	+		+	+				
Sobur, 2022	+	+	+		+	+		+		
Šrámová, 1992	+		+		+	+				
Stypułkowska-Misiurewicz, 2006	+		+		+	+				
Tilahun, 2012	+	+	+		+	+				
Tufa, 2020	+	+	+		+		+			
Watanabe, 2019	+	+				+			+	+
Wiktorczyk-Kapischke, 2022	+	+	+				+			
Zarchi, 2009	+	+	+		+					

Note. MALDI-TOF MS = Matrix Assisted Laser Desorption-Ionization Time of Flight Mass Spectrometry; Many studies identified microbes using polymerase chain reaction (PCR) to find specific genes such as mecA1, mecA2, blaCTX-M, blaOXA, blaNDM, AacA-D, ermA, tetK, tetM, vatA, mrsA, vatB, linA, blaZ, cat1, gyrA, grlA, dfrA, and rpoB genes. These were listed as “other,” though they are not the only “other” method used to identify microbes. See original manuscripts for greater depth.

* Methods not documented in the manuscript.

Only a small number of studies explicitly aimed to link arthropod-associated microbes with patients or healthcare-associated infections (HAI), including one study addressing dengue virus.^12^ Most investigations instead focused on describing the presence and diversity of microbe-carrying arthropods collected in HEs. Several of these targeted specific organisms of concern, such as foodborne pathogens and multidrug-resistant (MDR) bacteria, while a subset incorporated community-based collections (eg, homes, slaughterhouses, and residential facilities) for comparison.

A smaller group of studies concentrated on high-consequence pathogens such as *S. aureus*, *S. pneumoniae, S. pyogenes, S. agalactiae*, and selected MDR Gram-negatives (GNs). Additional work examined seasonal variation in arthropod abundance and carriage of *S. aureus*. Finally, two studies evaluated mosquito-borne viruses within HEs, underscoring the diversity of potential pathogens carried by arthropods.

### Outcomes

#### Microbial linkage between arthropods and patients

Six studies used methods designed to demonstrate microbial colonization or infection of both patients and arthropods captured in the hospital.^12–17^ However, only three were able to infer an association between arthropods and patients. Lima et al. cultured samples from patients and ants captured in a hospital in Brazil.^13^ Cultures from ants yielded 41 different species of bacteria and 18 from patients. Common isolates included Coagulase negative *Staphylococcus spp.* (both), *Acinetobacter baumannii* (both), *Acinetobacter lwoffii* (ant), *Staphylococcus aureus* (ant), and *Klebsiella pneumoniae* (patients). Though both sources were positive for similar bacteria, direct linkage was not demonstrated.

Fotedar and colleagues collected samples from both arthropods (houseflies) and patients with infected wounds (India, 1992).^14^ *Klebsiella* spp. was isolated from 36.7% of captured houseflies and 28.1% of sampled wound infections. Advanced methods to demonstrate genetic similarities were not available in 1991, limiting the ability to demonstrate a definite link. However, antibiograms for bacteria isolated demonstrated a high degree of resistance (82% in houseflies and 96.3% of *Klebsiella spp.* isolated from patients), while the antibiogram among controls showed only 8.7% of the samples were similarly resistant.

Almeida-Nunes et al. assessed healthcare transmission of dengue virus in Brazil.^12^ Four patients hospitalized more than 10 days before exhibiting symptoms and demonstrating positive serology were identified. Previous studies had already demonstrated transmission via needle stick injuries, blood transfusions, transplantation, and other mucosal exposures. The investigators found mosquitoes within the emergency department positive for dengue, as well as breeding sites and viable larvae. They concluded that the likely source of infection was from the mosquitoes in the hospital.

A study from South Africa by Cotton et al. was the first to genetically demonstrate similarity between cockroaches captured in a neonatal intermediate care unit and patient isolates.^15^ During an investigation of an outbreak of extended spectrum beta-lactamase (ESBL)-producing *Klebsiella pneumoniae*, the team observed cockroaches on “work surfaces, medical equipment such as incubators, in sharps containers, and even on infants.” Investigators collected environmental samples from the unit and nasal/rectal swabs from healthcare workers (possible carriers). The areas observed with cockroaches were positive for ESBL-producing *K. pneumoniae*, while the specimen from healthcare workers were negative. Bacterial isolates from cockroaches and patients demonstrated indistinguishable patterns on pulsed-field gel electrophoresis. The authors assert the evidence is not robust enough to prove a causal connection. However, the circumstantial evidence related to the outbreak, as well as negative cultures in controls, strongly support the likelihood of either direct (cockroaches crawling on infants) or indirect (cockroaches contaminating environmental surfaces) transmission via arthropod.

A similar study conducted by Hanrahan et al. identified a genetic association between cockroaches in an ICU and patients with HAI due to MDR *Enterobacter cloacae* (MRE).^17^ Upon identification of the outbreak, the team implemented robust infection prevention measures and screened all new and existing patients in the unit for MRE. They cultured environmental surfaces, multidose vials, protein supplements, and hands of healthcare workers (HCWs). They also required prolonged closure of rooms after terminal cleaning to ensure rooms were negative for MRE prior to housing a new patient. Environmental surfaces were culture positive for MRE, and hands of HCWs and medications/supplements were negative.

Cockroaches were implicated as possible sources of MRE after being observed in the ICU. As further evidence supporting this theory, rooms left empty that were initially negative for MRE would turn positive while still closed. This suggested contamination by a source other than healthcare workers and patients. Cockroaches caught in the ICU were positive for MRE, confirmed to be genetically similar to patient isolates and environmental samples on PFGE. Again, the study design did not permit a direct causal link between the cockroach and patient. However, the authors concluded cockroaches contributed to environmental contamination and cross-transmission to patients. Enhanced pest management resulted in the resolution of the outbreak.

Finally, Hassan et al. used advanced molecular analyses to demonstrate clear linkages between arthropods, hospital surfaces (HS), and patients (Pakistan, 2021).^16^ The investigators collected samples from surgical site infections (SSIs), hospital surfaces, and arthropods to isolate MDR *Enterobacteraciae* in a public hospital of Pakistan. They sought specific resistance genes, determined antibiotic sensitivity profiles, and conducted whole genome sequencing to identify specific genotypes of bacteria and phylogenetic relationships between isolates. They also used single nucleopeptide polymorphism (SNP) analysis to study transmission events. Though it was impossible to determine temporal precedence across the various samples, investigators did find a high degree of similarity and genetic linkages between surgical site infections, the environment, and arthropods. For example, “the majority of *E. coli* linkages occurred between arthropods and SSIs (*n* = 14) and, to a lesser extent, between HSs and SSIs (*n* = 4).”^16^ The authors acknowledge that the study was not designed to show causality, rather the interconnectedness of clinically relevant pathogens across the hospital. However, it is the most rigorous study to demonstrate the likelihood of arthropods as vectors of transmission to date.

#### Microbial carriage of arthropods in healthcare settings

The majority of studies that met inclusion criteria for this review only assessed carriage of arthropods. Supplemental Tables 2 and 3 detail the pathogens identified from the alimentary tract or external surfaces of arthropods captured in the HE ^2,12,15–43,45–56^ or in conjunction with a community control.^14,30,57–77^ All studies isolated microbes from arthropods. Only one study sought to isolate enteroviruses from cockroaches and failed to do so.^55^

Forty-five studies isolated *Staphylococcus* spp., nine of which isolated methicillin-resistant *Staphylococcus aureus* (MRSA).^17,25,27,35,36,48,53,75^ MRSA was isolated from cockroaches (German and American), various species of fly, and ants. Studies also examined multiple species of *Enterococcus* including clinically relevant *E. faecalis* and *E. faecium*.^13,39,42,63,64,66,73,75^ Numerous studies identified *Enterococcus* to the genus level but did not distinguish by species (Supplemental Table 2). These bacteria were isolated from ants, cockroaches, houseflies, moth flies, spiders, beetles, and wasps.

*Streptococcus* spp. were isolated from houseflies, cockroaches, ants, and moth flies.^2,22,28,32,34–40,45,46,54,54,57,58,62–64,66,73,78^ Once again, most were not identified to the species level. However, several studies isolated *S. pyogenes* from cockroaches and ants^34,39,63,71^ and *S. pneumoniae* from cockroaches.^73^ Supplemental Table 2 details other Gram-positive cocci (GPC) isolated from various arthropods. However, few are noted to be clinically relevant except as opportunistic pathogens in immunocompromised patients.

Not unexpectedly, cockroaches, moth flies, cigarette beetles, and ants were found to harbor the foodborne pathogen *Bacillus cereus*.^2,33,39,42,63,73^ Environmental and opportunistic pathogen, *Acinetobacter baumanii*, was isolated from houseflies, moth flies and ants.^2,13,13,24,45^ Additional species of *Acinetobacter* were also cultured including *A. lwolffii, A. calcoaceticus, and A. haemolyticus*.^2,13,41,45,76,77^ Each of these bacteria have been implicated in HAIs and can exhibit MDR.^79–82^ Other Gram positives isolated from ants, moth flies, and cockroaches included *Clostridium* spp. (*C. perfringens* isolated from ants),^39^ *Corynebacterium* spp.,^2,35,45,46,76,77^ *Nocardia* spp.,^43,45,52^ *Wolbachia* spp.,^33^ *Listeria monocytogenes*,^46^ and numerous other non-clinically relevant Gram positives.^45^

GNs were isolated from almost every study in this review and all studied arthropods except mosquitoes. *Pseudomonas* spp., including MDR strains, was isolated from cockroaches, ants, houseflies, moth flies, and cluster flies in thirty-six studies. Other GNs included *Enterobacter* spp. (*E. cloacae* and *E. aerogenes*), *Citrobacter* spp. (*C. freundii*) *Klebsiella* spp. (*K. pneumoniae, K. oxytoca*), *Serratia* spp. (*S. marcescens*, *S. liquefaciens*), *E. coli Salmonella* spp. and *Shigella* spp.^22,39,54,58,63,73^ Two other important environmental pathogens isolated included *Stenotrophomonas maltophilia* and *Burkholderia cepacia*.^2,13,20,42,43,57^ Flies, including cluster and moth flies, were the most common source of *Stenotrophomonas*, followed by ant and cockroach, while *Burkholderia* was isolated from ants.^13^ Many other GNs isolated from arthropods are listed in Supplemental Table 2.

Gram-negative cocci were not identified as abundantly as Gram-negative rods. However, *Neisseria* spp., including *N. meningitidis,* were found among ants and moth flies in this review,^2,13,39,46^ as were *Haemophilus* spp.^63,71^ There were additional Gram-variable bacteria isolated and many environmental bacteria less well-known in the healthcare environment.

Five studies isolated and identified viruses from mosquitoes in the HE and surrounding areas. Ehelepola et al. and Almeida-Nunes et al. captured mosquitoes positive for dengue viruses 1–4 in healthcare facilities in Sri Lanka and Brazil, respectively.^12,83^ Chiuya et al. identified several viruses from mosquitoes captured in their community controls in Kenya but only the Sindbis virus was identified when captured in the HE.^59^ Finally, Krokovsky et al found Zika and Chikungunya viruses among mosquitoes captured in HEs of Brazil.^51^

Lastly, 19 studies isolated and identified fungi and yeast from arthropods captured in their HEs (Supplemental Table 3). *Aspergillus* spp. (*A. flavus, A. fumigatus, A. niger*) were highly prevalent among cockroaches, flies, and ants.^21,26,28,30,35,45,49,50,55,64,66,67,71,76,84,85^ *Candida* spp., including *C. albicans, C. glabrata, C. parapsilosis*, and others were also identified in many of the same studies.

## Discussion

Arthropods in healthcare environments have received considerable attention over the past three decades. Despite the volume of studies highlighting the risk of transmission via arthropods, few have demonstrated a definitive link to HAIs. To date, no study has established temporal precedence (eg, arthropod exposure occurred before infection) between arthropod presence and patient infection. The few that reported associations between arthropods, patients, and the HE acknowledged the inability to infer causality.

Future research must move into controlled laboratory settings. While it is not possible to demonstrate direct transmission (in most reports, no arthropod is seen directly contaminating an open wound), the fact that arthropods carry these organisms and defecate in the HE is sufficient to infer causality. Experimental designs simulating HEs and susceptible hosts could clarify whether arthropods acquire and transmit unique pathogens.^86^

Across the studies reviewed, arthropods captured within HEs carried diverse and clinically relevant pathogens, often with microbial profiles distinct from those found in community or control settings, including MDR bacteria.^57,65,69,73^ The CDC recommend all facilities have an integrated pest management (IPM) plan,^5^ and the United States Environmental Protection Agency (EPA) provides resources and toolkits for implementation.^4^ While exclusion remains best practice, arthropods are ubiquitous and can persist even in facilities with active IPMs. IPMs should extend beyond exclusion to encompass risk management when arthropods are present. Sinks and drains in patient care areas warrant particular attention as potential arthropod entry points. These moist environments not only support biofilms containing clinically significant, MDR organisms but also provide ideal breeding sites for arthropods that can foster environmental contamination.^87–89^

While the potential for environmental contamination to contribute to HAIs has long been recognized, it has historically been underrepresented in both research and prevention efforts compared with other transmission routes.^90–93^ However, evidence from Hassan, Cotton, and Hanrahan et al. suggests that enhanced environmental cleaning is critical after any observation of active arthropods in patient care areas—particularly around high-touch surfaces and supplies used in wound care, invasive procedures, or care of high-risk patients (eg, baby bottles, suction controls, sterile supplies).^15–17^ The CDC’s *Antibiotic Resistance Threats in the United States* report reinforces the need for comprehensive environmental infection control, noting that drug-resistant organisms can survive and propagate in healthcare settings when such programs are insufficient.^94^

IPMs should also consider seasonal variations in arthropod activity^25,45^ and reflect this in facility risk assessments. Environmental disruptions such as construction can also precipitate infestations or heightened activity. Both internal and external environmental changes can drive arthropods into healthcare facilities.^95^

These recommendations apply globally, not only in low-and middle-income countries. IPs in all settings require evidence-based guidance to prevent pathogen transmission via arthropods. While the CDC’s *Guidelines for Environmental Infection Control* help mitigate this risk, evidence from developed countries also demonstrates potential for nosocomial transmission.^2,17,33,36–38,40–43,45,75–77,84^ Healthcare facilities should develop action plans for managing arthropod sightings and expand staff education on pest-related risks. Studies by Cotton and Hanrahan et al. revealed that healthcare personnel often observe arthropods in patient care areas without recognizing associated infection risks.^15,17^ Similarly, anecdotal experiences among the authors underscore the need for greater awareness. Arthropods in HE differ microbiologically from those in community environments, and this distinction must be communicated clearly to staff.

IPs and healthcare epidemiologists should consider arthropods as potential contributors to outbreaks, especially when a source is elusive. Genetic and phylogenetic analyses of arthropods, environmental samples, and patient isolates could clarify potential transmission pathways. While hand hygiene remains essential, arthropods may act as environmental reservoirs that facilitate indirect transmission.^15–17^ During outbreak investigations, capturing and testing arthropods should be considered, in collaboration with external entomologic or microbiologic partners.

### Limitations

Although none of the reviewed studies demonstrated a causal link between arthropods and patient infections, the weight of evidence supports their potential role in healthcare-associated transmission. We recognize the methodological limitations of existing studies but advocate for renewed attention and research investment in this domain. The consistent detection of clinically relevant and MDR microbes among healthcare-dwelling arthropods warrants further study and stronger guidance from regulatory and advisory agencies. We also recognize limitations within the methodology. Our study only included studies that were available in the English language. Despite this limitation, we found a highly representative number of articles (*n* = 73) from around the world. We also acknowledge that our review is at risk of publication bias. Only one of the studies that met our inclusion criteria reported negative findings. It is possible that research related to microbial transmission via arthropod vectors that failed to find an association was not published.

Finally, we acknowledge the possibility of unintended errors during data collation. However, given that the primary objective of this review was to provide a comprehensive overview of existing literature and highlight studies demonstrating a direct link between arthropods and nosocomial infection, minor inaccuracies in data extraction are unlikely to meaningfully affect the overall conclusions.

## Conclusions

This systematic review represents one of the most comprehensive examinations of arthropods’ roles in HAIs. At a minimum, arthropods serve as potential sources of environmental contamination. Because arthropods cannot be fully eliminated from healthcare settings, IP efforts must address multiple points in the chain of infection. These include eliminating open drains and plumbing sources, disrupting biofilms in moist breeding areas, and enhancing environmental cleaning of patient surroundings and high-touch surfaces, particularly during periods of increased arthropod activity. Through cross-disciplinary collaboration, rigorous research, and practical action, the healthcare community can better understand and mitigate the risks posed by arthropods in patient care environments.

## Supporting information

10.1017/ash.2025.10266.sm001 Stroever et al. supplementary material Stroever et al. supplementary material

## Supplementary material

The supplementary material for this article can be found at http://doi.org/10.1017/ash.2025.10266

## Prior presentation

The results of this study were presented during the poster sessions of SHEA Spring 2025.
