# Supplementary material for: From infestation to infection: a systematic review of arthropod-mediated microbial transmission in hospitals
Source: Antimicrob Steward Healthc Epidemiol. 2026 Jan 12;6(1):e16. doi: 10.1017/ash.2025.10266 (PMC12813724; doi:10.1017/ash.2025.10266)
Supplement: Stroever et al. supplementary material [file S2732494X25102660sup001.docx]

**Supplemental Table 1**. Arthropods examined for carriage, colonization, or transmission of microbes by study, *Genus* with *species* and lay terms identified

| **Arthropod group** | **Study** | **Genus & species (Lay term)** |
| --- | --- | --- |
| Ant | Aquino, 2013 | *Paratrechina longicornis* (Crazy ant) *Pheidole megacephala* (Big-headed ant) *Solenopsis saevissima* (Fire ant)  *Tapinoma melanocephalum* (Ghost ant) |
|  | Beatson, 1972 | *Monomorium pharaonis L* (Pharaoh ant) |
|  | Chadee ,1990 | *Monomorium pharaonic* (Pharaoh ant) *Solenopsis molestus* (Thief ant)  *Tapinoma sessile* (Odorous house ant) |
|  | DoNascimento, 2020 | *Brachymyrmex sp* (Rover ant)  *Linepithema humile* (Argentine ant) *Monomorium floricola* (Trailing/flower ant) *Monomorium pharaonis* (Pharaoh ant)  *Paratrechina longicornis* (Longhorn crazy ant) *Solenopsis saevissima* (Fire ant) *Tapinoma melanocephalum* (Ghost ant) *Tetramorium bicarinatum* (Tramp ant) *Wasmmania auropunctata* (Electric ant) |
|  | Frickmann, 2024 | *Lasius neglectus* (Garden ant) |
|  | Lima, 2013 | *Atta sp.* (Leafcutter ant)  *Camponotus vittatus* (Carpenter ant)  *Crematogaster victima* (Acrobat ant) *Solenopsis saevissima* (Fire ant) *Paratrechina fulva* (Tawny crazy ant) *Paratrechina longicornis* (Longhorn crazy ant)  *Pheidole sp*. (Big-headed ant) *Tapinoma melanocephalum* (Ghost ant) |
|  | Lise, 2006 | *Brachymyrmex sp (*Rover ant)  *Camponotus sp* (Carpenter ant) *Monomorium pharaonic* (Pharoah ant) *Paratrechina longicornis* (Longhorn crazy ant)  *Solenopsis saevissima* (Fire ant) *Solenopsis sp* (Fire ant) *Tapinoma melanocephalum* (Ghost ant) |
|  | Maximo, 2014 | *Brachymyrmex* (Rover ant)  *Dorymyrmex* (Pyramid ant) *Monomorium floricola* (Flower ant) *Paratrechina spp* (Crazy ant) *Pheidole* (Big-headed ant) |
|  | Oliveira, 2017 | *Crematogaster spp.* (Acrobat ant)  *Linepithema spp.* (Argentine ant)  *Pheidole spp.* (Big-headed ant) *Tapinoma melanocephalum* (Ghost ant) |
|  | Rodovalho, 2007 | *Camponotus vittatus* (Carpenter ant) *Tapinoma melanocephalum* (Ghost ant) |
|  | Rodríguez, 2016 | *Tapinoma melanocephalum* (Ghost ant) |
| Beetle | Watanabe, 2019 | *Lasioderma serricorne* (Cigarette beetle) |
| Cockroach | Abdolmaleki, 2019a | *Blattella germanica* (German cockroach)  *Periplaneta americana* (American cockroach) |
|  | Abdolmaleki, 2019b | *Blattella germanica* (German cockroach)  *Periplaneta americana* (American cockroach) |
|  | Adegoke, 2021 | *Periplaneta americana* (American cockroach) |
|  | Chehelgerdi, 2021 | *Blattella germanica* (German cockroach)  *Blatta orientalis* (Oriental cockroach)  *Periplaneta americana* (American cockroach) |
|  | Cotton, 2000 | *Blattella germanica* (German cockroach)  *Periplaneta americana* (American cockroach) |
|  | Davari, 2023 | *Blattella germanica* (German cockroach)  *Blatta orientalis* (Oriental cockroach)  *Periplaneta americana* (American cockroach) |
|  | Elgderi, 2006 | *Blattella germanica* (German cockroach) |
|  | Fakoorziba, 2014 | *Blattella germanica* (German cockroach)  *Periplaneta americana* (American cockroach) |
|  | Fotedar, 1991a | *Blattella germanica* (German cockroach) |
|  | Fotedar, 1991b | *Blattella germanica* (German cockroach) |
|  | Fotedar, 1992b | *Blattella germanica* (German cockroach) |
|  | Gliniewicz, 2003 | *Blattella germanica* (German cockroach) |
|  | Hanrahan, 2024 | Unspecified |
|  | Jalil, 2023 | *Blattella germanica* (German cockroach)  *Blatta orientalis* (Oriental cockroach)  *Periplaneta americana* (American cockroach) |
|  | Kassiri, 2014 | *Periplaneta americana* (American cockroach) |
|  | Khodabandeh, 2020 | *Periplaneta americana* (American cockroach) |
|  | Landolsi, 2022 | *Blattella germanica* (German cockroach)  *Blatta orientalis* (Oriental cockroach)  *Periplaneta americana* (American cockroach) |
|  | LeGuyader, 1989 | *Supella supellectilium* (Brown-banded cockroach) |
|  | Lemos, 2006 | *Periplaneta americana* (American cockroach) |
|  | Loucif, 2016 | *Blattella germanica* (German cockroach) |
|  | Madani, 2023 | *Blattella germanica* (German cockroach) |
|  | Mehainaoui, 2021 | *Blattella germanica* (German cockroach) |
|  | Memona, 2017 | *Blattella germanica* (German cockroach)  *Periplaneta americana* (American cockroach) |
|  | Menasria, 2014 | *Blattella germanica* (German cockroach) |
|  | Merad, 2023 | *Blattella germanica* (German cockroach)  *Periplaneta americana* (American cockroach) |
|  | Naher, 2018 | *Blattella germanica* (German cockroach) |
|  | Oliva, 2010 | *Blattella germanica* (German cockroach) |
|  | Pai, 2003 | *Blattella germanica* (German cockroach)  *Periplaneta americana* (American cockroach) |
|  | Pai, 2004 | *Blattella germanica* (German cockroach)  *Periplaneta americana* (American cockroach) |
|  | Prado, 2006 | *Periplaneta americana* (American cockroach) |
|  | Salehzadeh, 2007 | *Blattella germanica* (German cockroach) |
|  | Stypułkowska-Misiurewicz, 2006 | *Blattella germanica* (German cockroach) |
|  | Tilahun, 2012 | *Blattella germanica* (German cockroach) |
|  | Zarchi, 2009 | *Blattella germanica* (German cockroach)  *Periplaneta americana* (American cockroach) |
| Fly | Boiocchi, 2019 | *Diptera: Calliphoridae* (Blow fly) *Diptera: Dolichopodidae* (Long-legged fly) *Diptera: Fanniidae* (Lesser house fly) *Diptera: Muscidae* (House fly) *Diptera: Phoridae* (Scuttle fly) *Diptera: Psychodidae* (Moth fly/Drain fly) *Diptera: Sphaeroceridae* (Lesser dung fly) |
|  | Carramaschi, 2019 | *Malacophagomia filamenta* (Flesh fly) |
|  | Davari, 2012 | *Musca domestica* (House fly) |
|  | Davies, 2014 | *Musca domestica* (House fly) |
|  | Faulde, 2001 | *Pollenia rudis sensu stricto* (Cluster fly) |
|  | Faulde, 2013 | *Diptera: Clogmia albipunctata* (Moth fly) |
|  | Fotedar, 1992a | *Musca domestica* (House fly) |
|  | Fotedar, 1992c | *Musca domestica* (House fly) |
|  | Heiden, 2020 | *Musca domestica (*House fly) |
|  | Kassiri, 2015 | *Musca domestica* (House fly) |
|  | Monyama, 2023 | *Musca domestica* (House fly) |
|  | Nazari, 2017 | *Musca domestica* (House fly) |
|  | Rahuma, 2005 | *Musca domestica* (House fly) |
|  | Ranjbar, 2016 | *Musca domestica* (House fly) |
|  | Rupprecht, 2020 | *Clogmia albipunctata* (Moth fly) |
|  | Sobur, 2022 | *Musca domestica* (House fly) |
|  | Tufa, 2020 | Unspecified |
|  | Wiktorczyk-Kapischke, 2022 | *Calliphora* *vicina (*Blue bottle fly)  *Lucilia sericata* (Green bottle fly)  *Musca domestica* (House fly) |
| Mosquito | Almeida-Nunes, 2016 | *Aedes aegypti* (Yellow fever mosquito) |
|  | Chiuya, 2021 | *Aedes aegypti* (Yellow fever mosquito) *Anopheles spp*. (Malaria mosquito)  *Culex pipiens* (Common house mosquito) |
|  | Ehelepola, 2018 | *Aedes aegypti* (Yellow fever mosquito) |
|  | Krokovsky, 2022 | *Aedes aegypti* (Yellow fever mosquito) *Culex quinquefasciatus* (Southern house mosquito) |
| Multiple | Daniel, 1992 | *Anthomyidae gen. sp. (*Root-maggot flies)  *Araneus sp*. (Angulate & round-shouldered orb weavers spider)  *Blattella germanica* (German cockroach)  *Calliphora vicina* (Blue bottle fly)  *Coproica ferruginata (*Lesser dung fly)  *Coproica sp. 1 (*Lesser dung flies)  *Coproica sp. 2 (*Lesser dung flies)  *Coproica vagans (*Small dung fly)  *Culex pipiens molestus (*London Underground Mosquito)  *Drosophila busckii (*Fruit fly)  *Drosophila funebris* (Fruit fly)  *Drosophila immigrans* (Vinegar fly)  *Drosophila melanogaster* (Fruit fly)  *Fannia canicularis* (Little house fly)  *Fannia scalaris* (Latrine fly)  *Fannia sp*. (Small house fly)  *Hydrotaea ophyra sp.* (Dump fly)  *Hydrotaea aenescens* (Black dump fly)  *Hydrotaea ignava* (House fly)  *Lasius emarginatus* (ManhattAnt)  *Lasius n*i*ger* (Black garden ant)  *Lauxaniidae gen. sp*. (Lauxaniid flies)  *Leptocera sp. 1* (Lesser dung flies)  *Leptocera sp. 2* (Lesser dung flies)  *Limosina sp.* (Lesser dung flies)  *Liopiophila varipes* (Necrophagous fly)  *Lucilia ampullacea* (Streakless greenbottle fly)  *Lucilia caesar* (Common greenbottle fly)  *Lucilia sericata* (Common Green Bottle Fly or Sheep Blow Fly)  *Mitopus morio* (Harvestman spider)  *Musca domestica* (Housefly)  *Muscidae gen. sp*. (House flies and relatives)  *Muscina pabulorum* (Biting house fly)  *Muscina stabulans* (False stable fly)  *Mycetaulus bipunctatus* (Picture-winged fly)  *Nuctenea umbratica* (Walnut orb-weaver spider)  *Paravespula vulgaris* (Common wasp)  *Piophilidae gen. sp*. (Skipper flies)  *Protophormia terraenovae* (Blowfly)  *Sarcophagidae gen. sp*. (Flesh flies)  *Sepsis violacea* (Black scavenger fly)  *Sphaerocera sp*. (Lesser dung flies)  *Sphaeroceridae gen. sp*. (Lesser dung flies) *Steatoda bipunctata* (Rabbit hutch spider)  *Stomoxys calcitrans* (Stable fly)  *Syritta pipiens* (Thick-legged hoverfly)  *Tachinidae* (Tachinid flies)  *Tegenaria domestica* (Barn funnel weaver spider) *Tenebrio molitor* (Mealworm Beetle)  *Trachyopella sp*. (Lesser dung flies) |
|  | Hassan, 2021 | *Aglossa aglossalis* (Snout moth) *Blattella germanica* (German cockroach) *Blatta orientalis* (Oriental cockroach) *Lasius niger* (Black garden ant) *Musca domestica* (House fly) *Stegodyphus pacificus* (Velvet spider) |
|  | Kappel, 2013 | *Diptera: Unspecified* (True fly)  *Homoptera: Unspecified* (Aphid, whitefly, cicada)  *Hymenoptera: Unspecified* (Ant, bee, wasp) *Lepidoptera: Unspecified* (Moth, butterfly) *Orthoptera: Unspecified* (Grasshopper, cricket, locust) *Trichoptera: Unspecified* (Caddisfly) |
|  | Oliveira, 2014 | Unspecified (Ant)  Unspecified (Cockroach)  Unspecified (Fly)  Unspecified (Gnat)  Unspecified (Moth, butterfly)  Unspecified (Wasp) |
|  | Šrámová, 1992 | *Anthomyidae gen. sp.* (Root-maggot flies) *Aphidoidea gen. sp.* (Aphid)  *Araneus sp.* (Angulate & round-shouldered orb weavers spider)  *Athous niger* (Black click beetle)  *Blattella germanica* (German cockroach)  *Calliphora vicina* (Blue bottle fly)  *Coccinella septempunctata* (Seven-spot ladybird)  *Coproica ferruginata* (Lesser dung fly)  *Coproica sp. 1* (Lesser dung flies)  *Coproica sp. 2* (Lesser dung flies)  *Coproica vagans* (Small dung fly)  *Culex pipiens molestus* (London Underground Mosquito)  *Drosophila busckii* (Fruit fly)  *Drosophila funebris* (Fruit fly)  *Drosophila immigrans* (Vinegar fly)  *Drosophila melanogaster* (Fruit fly)  *Fannia canicularis* (Little house fly)  *Fannia scalaris* (Latrine fly)  *Fannia sp.* (Small house fly)  *Hemiptera* (True Bug)  *Hydrotaea ophyra sp.* (Dump fly)  *Hydrotaea aenescens* (Black dump fly)  *Hydrotaea ignava* (House fly)  *Lasius emarginatus* (ManhattAnt)  *Lasius niger* (Black garden ant)  *Lauxaniidae gen. sp.* (Lauxaniid flies)  *Leptocera sp. 1* (Lesser dung flies)  *Leptocera sp. 2* (Lesser dung flies)  *Limosina sp.* (Lesser dung flies)  *Liopiophila varipes* (Necrophagous fly)  *Lucilia ampullacea* (Streakless greenbottle fly)  *Lucilia caesar* (Common greenbottle fly)  *Lucilia sericata* (Common Green Bottle Fly or Sheep Blow Fly)  *Meligethes sp.* (Pollen beetle)  *Mitopus morio* (Harvestman spider)  *Musca domestica* (Housefly)  *Muscidae gen. sp.* (House flies and relatives)  *Muscina pabulorum* (Biting house fly)  *Muscina stabulans* (False stable fly)  *Mycetaulus bipunctatus* (Picture-winged fly)  *Nitidulidae gen. sp.* (Sap beetle)  *Nuctenea umbratica* (Walnut orb-weaver spider)  *Paravespula vulgaris* (Common wasp)  *Piophilidae gen. sp.* (Skipper flies)  *Protophormia terraenovae* (Blowfly)  *Sarcophagidae gen. sp.* (Flesh flies)  *Sepsis violacea* (Black scavenger fly)  *Sphaerocera sp.* (Lesser dung flies)  *Sphaeroceridae gen. sp.* (Lesser dung flies)  *Steatoda bipunctata* (Rabbit hutch spider)  *Stomoxys calcitrans* (Stable fly)  *Syritta pipiens* (Thick-legged hoverfly)  *Tachinidae* (Tachinid flies)  *Tegenaria domestica* (Barn funnel weaver spider)  *Tenebrio molitor* (Mealworm Beetle)  *Trachyopella sp.* (Lesser dung flies) |

**Supplemental Table 2.** Bacterial isolates identified in studies examining the carriage, colonization, or transmission of microorganisms via arthropods in healthcare and community-control settings.

| **Study ID** | **Gram positive bacteria** | | **Gram negative bacteria** | |
| --- | --- | --- | --- | --- |
| Abdolmaleki, 2019a | Methicillin resistant S*. aureus* (MRSA)  *S. aureus* (methicillin sensitive) | |  | |
| Abdolmaleki, 2019b | Methicillin resistant *S. aureus* (MRSA) | |  | |
| Adegoke, 2021 | *Staphylococcus* spp.  *Staphylococcus aureus* (methicillin resistance unspecified)  *Staphylococcus intermedius* | |  | |
| Beatson, 1972 | *S. epidermidis*  *S. aureus* (methicillin sensitive) *E. faecalis Streptococcus pyogenes Sarcina lutea Bacillus cereus*  *Bacillus pumilus Acinetobacter anitratus* | *Clostridium perfringens*  *Clostridium cochlearium* | *Klebsiella* spp. *Escherichia coli Salmonella dublin Proteus* spp. *Providencia* spp. *Pseudomonas aeruginosa*  *Pseudomonas fluorescens Neisseria sicca* | |
| Boiocchi, 2019 | *Staphylococcus* spp.*;*  *Staphylococcus* aureus without indication of methicillin resistance *Beta-hemolytic Streptococcus*, not speciated *Micrococcus* spp. *Bacillus* spp. *Clostridium* spp. | | *Enterobacter cloacae*  *Enterobacter* spp. *Citrobacter freundii Klebsiella pneumoniae*  *Klebsiella* spp. *Escherichia coli Leclercia* spp. *Pantoea* spp. | |
| Carramaschi, 2019 |  |  | *Raoultella ornithinolytica* | |
| Chadee, 1990 | *Enterococcus* spp. |  | *Klebsiella pneumoniae Proteus mirabilis Pseudomonas* spp. | |
| Chehelgerdi, 2021 | *Streptococcus pyogenes*  *Streptococcus pneumoniae*  *Streptococcus agalactiae* |  |  | |
| Cotton, 2000 |  |  | *Klebsiella pneumoniae* | |
| Daniel, 1992 | *Staphylococcus haemoluticus*  *Staphylococcus hominis Enterococcus* spp. *Acinetobacter calcoaceticus Corynebacterium* spp. |  | *Enterobacter cloacae*  *Enterobacter intermedius*  *Enterobacter agglomerans*  *Enterobacter aerogenes Citrobacter freundii*  *Citrobacter diversus*  *Citrobacter amalonaticus Klebsiella pneumoniae*  *Klebsiella oxytoca*  *Klebsiella ozaenae Serratia marcescens* | *Serratia fonticola Escherichia coli Hafnia alvei Providencia rettgeri Morganella morganii Pseudomonas aeruginosa*  *Pseudomonas fluorescens*  *Pseudomonas cepacia Flavobacter* spp.  *Acinetobacter calcoaceticus*  Sporulating microbes |
| Davari, 2023 | Coagulase negative *Staphylococcus Enterococcus* spp. *Bacillus* spp. *Acinetobacter* spp. | | *Enterobacter aerogenes Klebsiella* spp. *Escherichia coli Proteus* spp. *Pseudomonas aeruginosa* | |
| Davies, 2014 | *Staphylococcus aureus* (resistance pattern not specified) *Streptococcus* spp. *Bacillus lentus*  *Bacillus licheniformis* | *Bacillus pumilus*  *Bacillus subtilis*  *Clostridium* spp.  *Clostridium beijerinckii/butyrricum*  *Clostridum clostridioforme* | *Enterobacter cloacae*  *Enterobacter asburiae Citrobacter freundii Klebsiella pneumoniae*  *Klebsiella oxytoca* | *Escherichia coli*  *Escherichia hermannii*  *Pantoea* spp. *Raoultella terrigena* |
| DoNascimento, 2020 | *S. epidermidis*  *S. saprophyticus*  *S. aureus*  *S. epidermis*  *S. capitis* | *S. haemolyticus*  *S. choii*  *S. saprophyticus. Enterococcus* spp. *Bacillus* spp. | *Enterobacter* spp. *Klebsiella* spp. *Escherichia coli Pseudomonas aeruginosa* | |
| Elgderi, 2006 | *Streptococcus* spp. *Acinetobacter* spp. | | *Enterobacter cloacae*  *Enterobacter aerogenes Enterobacter gergoviae Enterobacter intermedius Citrobacter freundii*  *Citrobacter* spp.  *Citrobacter braakii Citrobacter youngae  Citrobacter amalonaticus  Klebsiella pneumoniae*  *Aeromonas hydrophila*  *Aeromonas* spp.  *Aeromonas caviae Pantoea* spp. *Buttiauxella agrestis*  *Klebsiella oxytoca*  *Klebsiella* spp.  *Klebsiella ornithinolytica* | *Serratia marcescens*  *Serratia liquefaciens*  *Serratia* spp.  *Serratia rubidaea  Serratia plymuthica Escherichia coli*  *Escherichia hermannii Hafnia alvei Proteus mirabilis*  *Proteus vulgaris Leclercia adecarboxylata; Leclercia* spp. *Morganella morganii*  *Morganella* spp. *Pseudomonas aeruginosa; Pseudomonas* spp. *Stenotrophomonas maltophilia* |
| Fakoorziba, 2014 | *S. saprophyticus Enterococcus* spp. *Bacillus* spp. *Acinetobacter* spp. Non-*Enterococcus* spp. | | *Enterobacter* spp. *Citrobacter* spp. *Klebsiella* spp. *Escherichia coli Shigella* spp. | *Proteus* spp. *Providencia* spp. *Morganella* spp. *Pseudomonas* spp. *Moraxella* |
| Faulde, 2001 | *Staphylococcus lugdunesis Bacillus* spp. | | *Pseudomonas aeruginosa Stenotrophomonas maltophilia  Erwinia amylovara*  *Flavobacterium odoratum* | |
| Faulde, 2012 | Coagulase-negative *Staphylococcus* spp. *Enterococcus* spp.  *E. casseliflavus Streptococcus* spp. *Micrococcus* spp. *Bacillus cereus*  *Bacillus* spp. *Acinetobacter baumanii*  *Acinetobacter calcoaceticus*  *Acinetobacter lwolffii*  *A. junii/johnsonii*  *A. haemolyticus Corynebacterium* spp. *Nocardia* spp. | | *Enterobacter cloacae*  *E. asburiae*  *E. sakazakii*  *E. aerogenes Citrobacter freundii*  *Citrobacter koseri Klebsiella pneumoniae*  *Klebsiella oxytoca Serratia marcescens*  *S. rubidaea*  *S. fonticola Escherichia coli Proteus mirabilis*  *Proteus* spp. *Providencia rettgeri Morganella morganii Yersinia frederiksenii Pseudomonas aeruginosa*  *P. stutzeri*  *P. putida*  *P. oryzihabitans*  *P. fleorescens Stenotrophomonas maltophilia*  *Aeromonas hydrophila* | *A. salmonicida*  *Alcaligenes faecalis*  *Alcaligenes* spp.  *A. denitrificans* Spp.*hingobacterium multivorum Neisseria* spp. *Burholderia cepacia,*  *Brevundimonas diminuta*  *Comamonas testosteroni Tsukamurella* spp.  *Photobacterium damsela Brevundimonas vesicularis Chryseomonas luteola Paecilomyces lilacinus Rhodococcus* spp.  *Streptomyces* spp.  *Myroides* spp.  *Psychrobacter phenylpyruvicus*  *Ralstonia pickettii*  *Shewaanella putrificans, Moraxella* spp.  *Vibrio parahaemolyticus* |
| Fotedar, 1991a | *S. epidermidis*  *S. aureus* (methicillin sensitive) *E. faecalis*  Listed as *“S. faecalis"  Streptococcus* spp.  Viridans group *streptococcus Micrococcus* spp. *Bacillus* spp. | | *Enterobacter* spp. *Klebsiella* spp. *Serratia* spp. *Escherichia coli Proteus* spp. *Pseudomonas aeruginosa* | |
| Fotedar, 1991b |  | | *Klebsiella pneumoniae*  *Klebsiella oxytoca*  *Klebsiella ozanae* | |
| Fotedar, 1992a |  | | *Klebsiella pneumoniae*  *Klebsiella oxytoca*  *Klebsiella ozanae*  *Klebsiella rhinoscleromatis* | |
| Fotedar, 1992c | *S. epidermidis*  *S. aureus* (methicillin sensitive)  *Staphylococcus* spp. *E. faecalis Streptococcus* spp.  Viridans group *Streptococcus*  *Micrococcus* spp. *Bacillus* spp. *Acinetobacter* spp. | | *Enterobacter* spp. *Klebsiella* spp. *Escherichia coli Proteus* spp. *Pseudomonas aeruginosa* | |
| Frickmann, 2024 | *Staphylococcus* spp. *Streptococcus* spp. *Micrococcus* spp. *Dermacoccus* spp. *Kocuria rhizophila  Paracoccus yeei  Weissella viridescens Bacillus* spp. *Acinetobacter baumanii Acinetobacter calcoaceticus Acinetobacter* spp.  *Acinetobacter haemolyticus Corynebacterium* spp. *Nocardia* spp. *Aneurinibacillus* spp. *Arthrobacter* spp. (can show coccoid forms in some conditions) *Brevibacillus brevis  Brevibacterium* spp. | *Cellulosimicrobium cellulans Fictibacillus arsenicus*  *Glutamibacter protophormiae  Glutamibacter* spp. *Lactobacillus* spp. *Leucobacter denitrificans  Lysinibacillus pakistanensis  Microbacterium* spp.*. Paenarthrobacter histidinolovorans  Paenarthrobacter* spp. *Paenibacillus* spp. *Pseudarthrobacter polychromogenes  Pseudarthrobacter* spp. *Streptomyces* spp. [filamentous (actinomycetes)] *Tsukamurella* spp. *(filamentous) Weissella viridescens* (can be Gram positive cocci) | *Citrobacter freundii Serratia marcescens*  *Serratia ficaria  Serratia rubidaea  Leclercia adecarboxylata Pseudomonas* spp. *Pantoea agglomerans Lelliottia* spp. *Ewingella americana  Moraxella* spp. (can also appear as coccobacilli) *Morganella morganii  Ochrobactrum tritici  Rhizobium* spp. | |
| Gliniewicz, 2003 | Methicillin resistant *S. aureus* (MRSA)  *S. epidermidis*  *S. equorum*  *S. hominis Enterococcus avium Enterococcus durans Streptococcus oestibularis Streptococcus salivarius*  *Micrococcus luteus* | | *Enterobacter cloacae Citrobacter freundii Klebsiella oxytoca Serratia marcescens Pseudomonas aeruginosa* | |
| Hanrahan, 2024 | Methicillin resistant *S. aureus* (MRSA)  *S. aureus* (methicillin sensitive)  *Coagulase-negative staphylococcal* spp. | | *Enterobacter* spp.  Multidrug resistant *Enterococcus* spp. Multidrug resistant *Pseudomonas* spp. | |
| Hassan, 2021 |  | | *Enterobacter cloacae Klebsiella pneumoniae Escherichia coli* | |
| Heiden, 2020 | *Acinetobacter baumanii* | | *Enterobacter cloacae Citrobacter freundii Klebsiella oxytoca Escherichia coli Pseudomonas aeruginosa Raoultella ornithinolytica* | |
| Jalil, 2023 | Coagulase negative *Staphylococcus* spp. *Enterococcus* spp. *Bacillus* spp. *Acinetobacter* spp. | | *Klebsiella* spp. *Escherichia coli Proteus* spp. *Pseudomonas aeruginosa* | |
| Kappel, 2013 | Coagulase-negative *staphylococci Micrococcus* spp. *Bacillus* spp. *Nocardia cyriacigeorgica* | | *Enterobacter gergoviae Proteus mirabilis Pseudomonas* spp. | |
| Kassiri, 2014 | *Staphylococcus* spp. *Streptococcus* spp. | | *Klebsiella* spp. *Escherichia coli Proteus* spp. *Pseudomonas* spp. | |
| Landolsi, 2022 |  | | *Enterobacter cloacae Citrobacter freundii*  *Citrobacter gillenii*  *Citrobacter sedlakii Klebsiella pneumoniae Serratia marcescens* | *Serratia rubidaea Escherichia coli Providencia rettgeri Leclercia adecarboxylata Pantoea* spp. |
| LeGuyader, 1989 | *S. aureus* (methicillin sensitive) *Acinetobacter calcoaceticus* | | *Enterobacter cloacae*  *Enterobacter sakazakii*  *Enterobacter agglomerans*  *Enterobacter amnigenus*  *Enterobacter aerogenes Citrobacter freundii*  *Citrobacter diversus Klebsiella pneumoniae*  *Klebsiella oxytoca Serratia marcescens*  *Serratia liquefaciens Escherichia coli*  *Escherichia adecarboxylata Proteus mirabilis* | *Kluyevera* spp. *Leclercia adecarboxylata Pseudomonas aeruginosa*  *Pseudomonas maltophilia*  *Pseudomonas* spp.  *Pseudomonas paucimobilis*  *Pseudomonas fluorescens*  *Pseudomonas stutzeri Aeromonas hydrophila Alcaligenes faecalis* |
| Lima, 2013 | *S. epidermidis*  *S. aureus* (methicillin sensitive)  *Staphylococcus* spp. *E. faecalis*  *E. faecium Acinetobacter baumanii*  *Acinetobacter lwolffii* | | *Enterobacter cloacae*  *Enterobacter aerogenes Klebsiella pneumoniae Serratia marcescens Escherichia coli Proteus mirabilis Providencia stuartii* | *Kluyevera* spp. *Pseudomonas aeruginosa Stenotrophomonas maltophilia  Pantoea agglomerans Burkholderia cepacia Neisseria meningitidis* |
| Lise, 2006 | *S. saprophyticus*  *Staphylococcus* spp.  *S. intermedius Enterococcus* spp. *Streptococcus agalactiae*  *Streptococcus bovis Acinetobacter* spp. | *Acinetobacter haemolyticus Corynebacterium* spp.  *C. diphtheriae*  *C. jeikeium Aeskovia* spp. *Listeria monocytogenes*  *Planococcus* spp. | *Pseudomonas luteola Sphingobacterium* spp. *Sphingomonas paucimobilis Neisseria* spp. | |
| Loucif, 2016 |  | | *Enterobacter cloacae*  *Enterobacter kobei Citrobacter freundii*  *Citrobacter koseri*  *Citrobacter amalonaticus*  *Citrobacter farmeri Klebsiella oxytoca* | |
| Máximo, 2014 | *S. epidermidis*  *Staphylococcus* spp. *Streptococcus* spp. *Micrococcus luteus Bacillus* spp. *Listeria* spp.  *Arcanobacterium* spp. | | *Klebsiella* spp. *Proteus* spp. *Pseudomonas aeruginosa* | |
| Mehainaoui, 2021 | *S. equorum*  *S. saprophyticus*  *Staphylococcus cohnii*  *Staphylococcus hominis*  *Staphylococcus succinus Enterococcus avium*  *Enterococcus asini*  *Enterococcus casseliflavus* | | *Citrobacter koseri*  *Citrobacter amalonaticus*  *Citrobacter braakii*  *Citrobacter youngae Klebsiella oxytoca Serratia marcescens*  *Serratia liquefaciens Kluyevera intermedia Leclercia adecarboxylata* | *Morganella morganii Pseudomonas aeruginosa*  *Pseudomonas azotoformans Stenotrophomonas maltophilia  Lysinibacillus sinduriensis*  *Pseudocitrobacter faecalis*  *Pseudocitrobacter vendiensis* |
| Memona, 2017 | *S. epidermidis*  *S. aureus* (methicillin sensitive) *E. faecalis Streptococcus pneumoniae Bacillus cereus* | | *Enterobacter cloacae*  *Enterobacter aerogenes Klebsiella pneumoniae Escherichia coli Salmonella typhimurium* | *Shigella dystenteriae Proteus mirabilis*  *Proteus vulgaris Pseudomonas aeruginosa* |
| Menasria, 2014 | *S. aureus* (methicillin sensitive)  *Staphylococcus* spp.  Non-pathogenic *Staphylococcus* | | *Enterobacter cloacae*  *Enterobacter* spp.  *Enterobacter aerogenes Citrobacter freundii Klebsiella pneumoniae* | *Serratia marcescens*  *Serratia* spp. *Pseudomonas aeruginosa Pseudomonas* spp. *Pantoea* spp. |
| Monyama, 2023 | *Enterococcus* spp. *Streptococcus* spp. *Acinetobacter* spp. | | *Enterobacter* spp. *Klebsiella* spp. *Serratia* spp. *Escherichia coli Shigella* spp. *Proteus* spp. *Providencia* spp. *Morganella* spp. *Moellerella* spp. *Pseudomonas* spp. *Wohlfahrtiimonas* spp. *Dysgonomonas* spp. | *Ignatzscheneria* spp.  *Suttonella* spp.  *Lactococcus* spp.  *Koukoulia* spp.  *Lactobacillus* spp.  *Weissella* spp.  *Myroides* spp.  *Caulobacter* spp.  *Vagococcus* spp.  *Coxiella* spp.  *Leuconostoc* spp. |
| Naher, 2018 | *S. epidermidis*  *S. aureus* (methicillin sensitive) *E. faecalis Streptococcus pyogenes Bacillus cereus* | | *Enterobacter* spp.*. Klebsiella* spp. *Escherichia coli Salmonella* spp. *Shigella* spp. | *Proteus* spp. *Pseudomonas aeruginosa Haemophilus* spp. |
| Nazari, 2017 | *S. aureus* (methicillin sensitive)  Coagulase negative *Staphylococcus Enterococcus* spp. *Bacillus* spp. | | *Enterobacter* spp.*. Citrobacter* spp.*. Klebsiella* spp. *Escherichia coli Proteus* spp. *Pseudomonas* spp. | |
| Oliva, 2010 | *S. epidermidis*  *S. aureus* (methicillin sensitive) *Enterococcus* spp. *Acinetobacter calcoaceticus* | | *Enterobacter cloacae*  *Enterobacter aerogenes, Enterobacter agglomerans Citrobacter freundii*  *Citrobacter diversus Klebsiella pneumoniae*  *Klebsiella oxytoca* | *Serratia marcescens*  *Escherichia coli Proteus mirabilis*  *Proteus vulgaris Providencia stuartii Pseudomonas aeruginosa Alcaligenes faecalis* |
| Oliveira, 2014 | Methicillin resistant *S. aureus* (MRSA)  *S. aureus* (methicillin sensitive) | |  |  |
| Oliveira, 2017 | Methicillin resistant *S. aureus (MRSA)*  *S. epidermidis*  *S. aureus* (methicillin sensitive)  *S. saprophyticus*  Coagulase negative *Staphylococcus* | | *Enterobacter* spp. *Citrobacter freundii Klebsiella oxytoca*  *Klebsiella* spp. *Escherichia coli Hafnia* spp. *Yersinia entercolitica* | |
| Pai, 2003 |  | | *Mycobacterium xenopi*  *Mycobacterium gordonae* I  *Mycobacterium haemophilum*  *Mycobacterium fortuitium*  *Mycobacterium avium*  *Mycobacterium kansasii* | |
| Pai, 2004 | Methicillin resistant *S. aureus* (MRSA)  *S. epidermidis*  *S. aureus* (methicillin sensitive) *Enterococcus* spp. *Streptococcus* spp. *Bacillus* spp. *Acinetobacter* spp. *Corynebacterium* spp. | | *Enterobacter cloacae*  *Enterobacter* spp.  *Enterobacter agglomerans*  *Enterobacter odorifera*  *Enterobacter aerogenes*  *Enterobacter gergoviae*  *Enterobacter dissolvens  Citrobacter freundii*  *Citrobacter diversus Klebsiella pneumoniae*  *Klebsiella oxytoca*  *Klebsiella* spp. *Serratia marcescens*  *Serratia odorifera* | *Serratia rubidaea Escherichia coli Hafnia alvei Proteus mirabilis*  *Proteus vulgaris Pseudomonas aeruginosa*  *Pseudomonas mallei*  *Pseudomonas cepacia  Sphingobacterium mizutae Pasteurella canis Oligella urethralis*  *Chromobacterium* spp. |
| Prado, 2006 | Coagulase negative *Staphylococci* spp. |  | *Enterobacter cloacae*  *Enterobacter aerogenes*  *Enterobacter gergoviae  Klebsiella pneumoniae Serratia marcescens*  *Serratia* spp. *Hafnia alvei* | |
| Rahuma, 2005 | *Staphylococcus* spp. *Streptococcus* spp. | | *Enterobacter* spp. *Citrobacter* spp. *Klebsiella* spp. *Serratia* spp. *Escherichia coli Salmonella* spp. *Shigella sonnei Proteus* spp. | *Providencia* spp. *Yersinia entercolitica Edwardsiella tarda*  *Erwinia* spp.  *Aeromonsas* spp.*,  Xanthomonas* spp.  *Weeksella zoohlecum*  *Pasteurella* spp. |
| Ranjbar, 2016 |  | | *Klebsiella pneumoniae* | |
| Rodovalho, 2007 | Coagulase-positive *Staphylococcus* spp.  Coagulase-negative *Staphylococcus* spp. | | Coliforms  Gram negative bacteria not further identified | |
| Rodríguez, 2016 | *Staphylococcus* spp. *Enterococcus* spp. *Streptococcus* spp. *Micrococcus* spp. *Bacillus* spp. | | *Enterobacter* spp.  *E. intermedius  Salmonella* spp. *Shigella* spp. | |
| Rupprecht, 2020 | *S. epidermidis E. faecium Bacillus cereus*  *Bacillus* spp.  *B. megatariume*  *B. thuringienses* | | *Citrobacter freundii Escherichia coli Pseudomonas aeruginosa; Pseudomonas* spp.  *P. mosselii*  *P. nitroreducen*  *P. putida* | *Stenotrophomonas maltophilia Chryseobacterium indologenses*  *Lysinibacillus* spp.*haericus*  *Achromobacter xylosoxidans Advenella* spp. |
| Salehzadeh, 2007 | *Staphylococcus* spp. *Enterococcus* spp. *Streptococcus* spp.  *Streptococcus* (group A) | | *Enterobacter* spp.*. Klebsiella* spp. *Escherichia coli Shigella* spp. *Pseudomonas* spp. *Haemophilus* spp. | |
| Sobur, 2022 | Methicillin resistant *S. aureus* (MRSA)  *S. aureus* (methicillin sensitive) | |  | |
| Šrámová, 1992 | *S. hominis*  *S. haemolyticus Enterococcus* spp. *Acinetobacter calcoaceticus*  *Acinetobacter* spp. *Corynebacterium* spp. Spore-forming bacteria | | *Enterobacter cloacae*  *Enterobacter* spp.  *E. agglomerans*  *E. aerogenes*  *E. intermedius Citrobacter freundii*  *Citrobacter diversus Klebsiella pneumoniae*  *Klebsiella ozaenae* | *Serratia marcescens*  *Serratia fonticola Escherichia coli Hafnia alvei Providencia rettgeri Morganella morganii Pseudomonas* spp.  *Pseudomonas cepacia Flavobacterium* spp. |
| Stypułkowska-Misiurewicz, 2006 | *S. epidermidis*  *S. equorum*  *S. hominis E. avium*  *E. durans S. oestibularis*  *S. salivarius Micrococcus luteus* | | *Enterobacter cloacae Citrobacter freundii Klebsiella oxytoca Serratia marcescens Pseudomonas aeruginosa*  *P. putida* | |
| Tilahun, 2012 | *S. aureus* (methicillin sensitive) Non-Group A *Streptococcus* spp. *Acinetobacter* spp. | | *Enterobacter cloacae*  *Enterobacter aeruginosa Citrobacter* spp.  *Citrobacter diversus Klebsiella pneumoniae*  *Klebsiella oxytoca* | *Klebsiella ozaenae Escherichia coli Salmonella* spp. *Shigella flexneri Providencia rettgeri Pseudomonas aeruginosa* |
| Tufa, 2020 | *Acinetobacter* spp. | | *Enterobacter* spp. *Citrobacter* spp. *Klebsiella* spp. *Serratia marcescens*  *Serratia liquefaciens Escherichia coli Hafnia alvei Proteus* spp. *Providencia* spp. *Kluyevera* spp. | *Leclercia adecarboxylata Moellerella wisconsensis Aeromonas hydrophila Pantonea agglomerans Lelliottia* spp. *Cedecea davisae*  *Raoultella* spp.  *Comamonas testosterone*  *Rahnella aquatilis*  *Yokenella regensburgei* |
| Watanabe, 2019 | *Bacillus cereus*  *B. subtilis Wolbachia* spp. | |  | |
| Wiktorczyk-Kapischke, 2022 | Methicillin resistant *S. aureus* (MRSA) *E. faecalis*  *E. faecium*  *Enterococcus gallinarum*  *Enterococcus casseliflavus*  *Enterococcus hirae* | | *Enterobacter cloacae*  *Enterobacter hormaechei Citrobacter freundii Klebsiella pneumoniae*  *Klebsiella oxytoca*  *Klebsiella varicola Serratia marcescens*  *Serratia liquefaciens Escherichia coli*  *Escherichia hermannii Hafnia alvei Proteus mirabilis*  *Proteus vulgaris*  *Proteus rettgeri*  *Proteus hausei* | *Providencia stuartii*  *Providencia rettgeri*  *Providencia alcalifaciens Leclercia adecarboxylata Morganella morganii Moellerella wisconsensis Bordatella trematum Plesiomonas shigelloides Pantoea agglomerans Wohlfahrtiimonas chitiniclastica Lelliottia* spp. *Raoultella ornithinolytica*  *Raoultella planticola* |
| Zarchi, 2009 | *S. epidermidis*  *S. aureus* (methicillin sensitive)  *S. saprophyticus Streptococcus* spp.  Strep Group D  Strep non group D  Strep non group A and B *Bacillus* spp. | | *Enterobacter cloacae*  *Enterobacter aerogenes Citrobacter freundii*  *Citrobacter diversus Klebsiella pneumoniae*  *Klebsiella oxytoca Escherichia coli Proteus mirabilis*  *Proteus vulgaris Morganella morganii Pseudomonas aeruginosa Edwardsiellae trada* | |

**Supplemental Table 3.** Viral, fungal, and yeast isolates identified by studies examining the carriage, colonization, or transmission of microorganisms via arthropods in healthcare and community-control settings.

| **Study ID** | **Viruses** | **Fungi/Yeast** | |
| --- | --- | --- | --- |
| Almeida-Nunes, 2016 | Dengue virus 1-4 |  | |
| Aquino, 2013 |  | *Aspergillus flavus*  *Scolecobasidium* spp  *Purpureocillium lilacinum*  *Aspergillus* spp*.*  *Cladosporium* spp.  *Fusarium* spp*.*  *Mucor* spp.  *Rhizopus* spp*.*  *Alternaria* spp. | *Verticillium* spp.  *Cunninghamella* spp*.*  *Cunninghamella echinulate*  *Penicillium* spp*.*  *Acremonium* spp.  *Fusarium solani*  *Scolecbasidium* spp.  *Rhodotorula* spp. |
| Chiuya, 2021 | Identified in **community setting**:  Flavivirus  Alphavirus  Phlebovirus Orthobunya-virus  Nairovirus  Thogotovirus  Identified in **healthcare** setting:  Sindbis virus |  | |
| Daniel, 1992 |  | Reported, but unspecified | |
| Davari, 2012 |  | *Aspergillus flavus*  *Aspergillus niger*  *Penicillium* spp.  *Fisarium* spp.  *Micro*spp.*ori gypseum*  *Alternria* spp. | |
| Ehelepola, 2018 | Dengue virus |  | |
| Fotedar, 1991a |  | *Candida* spp. *Rhizomes* spp. *Mucor* spp. *Alternaria* spp. | *Aspergillus niger Aspergillus flavus Aspergillus* spp. *Candida* spp. |
| Fotedar, 1992b |  | *Rhizopus Aspergillus niger Aspergillus flavus Aspergillus fumigatus* | *Alternaria* spp. *Mucor* spp. *Candida* spp. |
| Fotedar, 1992c* |  | *Aspergillus niger*  *Geotrichum* spp. *Candida* spp. | |
| Frickmann, 2024 |  | *Aspergillus* spp. Yeasts were identified, but were not specified. | |
| Kassiri, 2015 |  | *Aspergillus flavus*  *Aspergillus niger*  *Aspergillus fumigatus*  *Aspergillus terreus*  *Rhizopus* spp.  *Penicillium* spp.  *Mucorales* spp.  *Alternaria* spp.  *Paecilomyces* spp.  *Mycelia sterilia*  *Chrysosporium* spp.  *Monilia* spp.  *Geotrichum* spp.  *Tricho*spp.*oron* spp.  *Rhodotorula* spp. | *Zygosaccharomyces* spp.  *Kloeckera apiculata/apis*  *Debaromyces polymorphus*  *Aureobasidium* spp.  *Candida albicans*  *Candida parapsilosis*  *Candida* spp. *Candida famata*  *Candida colliculosa*  *Candida lambica*  *Candida guilliermondii*  *Candida catenulate*  *Candida lusitaniae,* |
| Khodabandeh, 2020 |  | *Aspergillus niger*  *Penicillium* spp.  *Rhizopus* spp.  *Mucor* spp*.,  Candida albicans*  *Candida glabrata* | *Candida* spp. *Candida krusei*  *Candida kluyveri*  *Candida viswanathii*  *Candida tropicalis* |
| Krokovsky, 2022 | Zika  Chikungunya  Dengue |  | |
| Lemos, 2006 |  | *Aspergillus flavus*  *Aspergillus fumigatus*  *Aspergillus* spp.  *Penicillium* spp.  *Alternaria* spp.  *Curularia* spp.  *Beauveria* spp.  *Micelia serilia* spp.  *Geotrichum* spp.  *Oidodendrum* spp. | *Pullularia* spp.  *Torulopsis* spp.  *Trichosporon* spp.  *Candida albicans*  *Candida parapsilosis*  *Candida glabrata*  *Candida* spp. *Candida pseudotropicalis* |
| Madani, 2023 |  | *Pathogenic fungi Pichia kudriavzevii*  *Pichia kluyveri*  *Aspergillus niger*  *Penicillium italicum* | *Mucor plumbeus*  *Rhizopus oryzae Candida glabrata*  *Candida* spp. *Candida viswanathii* |
| Merad, 2023 |  | *Rhizopus* spp.  *Aspergillus niger*  *Luchtheimia* spp.  Non-identified mycelium  *Aspergillus flavus* | *Penicillium* spp.  *Rhodotorula* spp.  *Cryptococcus humicola*  *Paecilmyces* spp. *Candida* non-albicans spp. |
| Naher, 2018 |  | Unspecified fungi and parasites |  |
| Oliva, 2010 |  | *Aspergillus* spp.  *Mucor* spp.  *Rizopus* spp. |  |
| Pai, 2004 |  | *Hansenula anomala*  *Penicillium specie*  *Aspergillus flavus*  *Mucor* spp.  *Cladosporium* spp.  *Absidia* spp. *Candida parapsilosis*  *Candida glabrata*  *Candida* spp. | *C. guilliermondii*  *C. krusei*  *C. lambica*  *C. lusitaniae*  *C. pintolopesii*  *C. rugosa*  *C. tropicalis*  *C. sake* |
| Prado, 2006 |  | Filamentous fungi Unspecified yeast |  |
| Rupprecht, 2020 |  | *Candida albicans* |  |
| Salehzadeh, 2007 |  | *Mucor* spp.  *Aspergillus niger*  *Rhizopus* spp.  *Penicillium* spp.  *Aspergillus fumigans Candida* spp. |  |
| Šrámová, 1992 |  | Fungi (not specified) |  |

*Fotedar et al (1992) Also found parasites: Endolimax nana, Iodamoeba buestchlii, Entamoeba coli, Entamoeba histolytica
